# Sphingosine-1-phosphate in Endothelial Cell Recellularization Improves Patency and Endothelialization of Decellularized Vascular Grafts In Vivo

**DOI:** 10.3390/ijms20071641

**Published:** 2019-04-02

**Authors:** Kai Hsia, Chih-Hsun Lin, Hsin-Yu Lee, Wei-Min Chen, Chao-Ling Yao, Chien-Chin Chen, Hsu Ma, Shyh-Jen Wang, Jen-Her Lu

**Affiliations:** 1 Department of Life Science, National Taiwan University, Taipei 10617, Taiwan; hkay1008@gmail.com (K.H.); hsinyu@ntu.edu.tw (H.-Y.L.); b99b01070@ntu.edu.tw (W.-M.C.); 2 Department of Pediatrics, Taipei Veterans General Hospital, Taipei 11217, Taiwan; 3 Division of Plastic Surgery, Department of Surgery, Taipei Veterans General Hospital, Taipei 11217, Taiwan; chlin12@vghtpe.gov.tw (C.-H.L.); sma@vghtpe.gov.tw (H.M.); 4 Department of Surgery, School of Medicine, National Yang-Ming University, Taipei 11221, Taiwan; 5 Department of Chemical Engineering and Materials Science, Graduate School of Biotechnology and Bioengineering, Yuan Ze University, Chung-Li, Taoyuan City 32003, Taiwan; d897601@alumni.nthu.edu.tw; 6 Department of Pathology, Ditmanson Medical Foundation Chia-Yi Christian Hospital, Chiayi 600, Taiwan; hlmarkc@gmail.com; 7 Department of Cosmetic Science, Chia-Nan University of Pharmacy and Science, Tainan 717, Taiwan; 8 Department of Surgery, medicine & Pediatrics, School of Medicine, National Defense Medical Center, Taipei 11490, Taiwan; 9 Division of Experimental Surgery, Department of Surgery, Taipei Veterans General Hospital, Taipei 11217, Taiwan; wangsj@vghtpe.gov.tw; 10 Department of Pediatrics, School of Medicine, National Yang-Ming University, Taipei 11221, Taiwan

**Keywords:** sphingosine-1-phosphate, vascular graft, endothelial cell, thrombosis, syndecan-1

## Abstract

Background: S1P has been shown to improve the endothelialization of decellularized vascular grafts in vitro. Here, we evaluated the potential of tissue-engineered vascular grafts (TEVGs) constructed by ECs and S1P on decellularized vascular scaffolds in a rat model. Methods: Rat aorta was decellularized mainly by 0.1% SDS and characterized by histology. Rat ECs, were seeded onto decellularized scaffolds, and the viability of the ECs was evaluated by biochemical assays. Then, we investigated the in vivo patency rate and endothelialization for five groups of decellularized vascular grafts (each *n* = 6) in a rat abdominal aorta model for 14 days. The five groups included (1) rat allogenic aorta (RAA); (2) decellularized RAA (DRAA); (3) DRAA with S1P (DRAA/S1P); (4) DRAA with EC recellularization (DRAA/EC); and (5) DRAA with S1P and EC recellularization (DRAA/EC/S1P). Results: In vitro, ECs were identified by the uptake of Dil-Ac-LDL. S1P enhanced the expression of syndecan-1 on ECs and supported the proliferation of ECs on decellularized vascular grafts. In vivo, RAA and DRAA/EC/S1P both had 100% patency without thrombus formation within 14 days. Better endothelialization, more wall structure maintenance and less inflammation were noted in the DRAA/EC/S1P group. In contrast, there was thrombus formation in the DRAA, DRAA/S1P and DRAA/EC groups. Conclusion: S1P could inhibit thrombus formation to improve the patency rate of EC-covered decellularized vascular grafts in vivo and may play an important role in the construction of TEVGs.

## 1. Introduction

There is a clinical need for tissue-engineered vascular grafts (TEVGs) in the cardiac field for peripheral bypass surgery, shunt surgery, microsurgery and pediatric vascular surgery. In autologous grafts, there are problems with the shortage or poor quality of patient vessels [1]. In allogenic or xenogenic grafts, immune reactions will be elicited after implantation that can lead to thrombosis and occlusion [2]. Tissue engineering techniques for developing vascular grafts incorporate scaffolds, cells and growth signals to create biomimetic tubular constructs, which are expected to overcome these problems [3]. Ideal TEVGs should provide biocompatibility, adequate endothelialization, cellular repopulation, compliance matching, biodegradability and tissue remodeling [4,5].

Recently, decellularized biological scaffolds have gained attention in the development of TEVGs. Decellularized vascular grafts have several advantages for clinical applications. First, these grafts are composed of natural extracellular matrix (ECM), mainly collagen, elastin and glycosaminoglycan (GAG) and other macromolecules [6]. The microenvironment of ECM is crucial for cell activity, and cell-scaffold “cross-talk” can initiate cell signaling, mitosis and differentiation. The niche could also enable tissue remodeling and regeneration [7]. Second, the collagen and elastin fibers of decellularized vascular grafts could provide elasticity to withstand pulsatile blood flow within a physiological range. Of these ECM components, collagen is responsible for the retention of tensile strength, elastin fibers maintain the elastic properties of the scaffold, and GAGs provide viscoelasticity [8,9]. Third, because most xenoantigens are removed, the decellularized xenogenic grafts serve as an abundant resource for vascular tissue free of the threat of acute rejection [5].

In our previous work, we demonstrated that S1P, a lipid mediator, could reduce platelet aggregation, inhibit thrombosis formation and improve the endothelialization of recellularized umbilical vein scaffolds in vitro. We also identified that S1P could reduce syndecan-1 (SDC-1) shedding from the endothelial cell surface and thus inhibit platelet adhesion on decellularized vascular grafts. SDC1 is one of the major heparan sulfate proteoglycans on EC with potent antithrombotic action and shedding from EC was one of the components in the thrombi found in animals with bacterial infection. S1P protects SDC-1 from shedding and makes it useful in construction of TEVGs which could resist thrombosis [10].

In this study, we hypothesized that S1P could further enhance patency and endothelialization and of decellularized vascular grafts in vivo. First, we prepared decellularized rat aortic scaffolds mainly by 0.1% SDS and characterized by histology. Rat ECs, isolated from the rat abdominal aorta, were cultured in vitro and the expression of SDC-1 in ECs either with or without S1P was checked. Then, the ECs were seeded on the decellularized scaffolds, and the viability of the ECs was evaluated by biochemical assays. We investigated the performance of decellularized vascular grafts incorporated with S1P in an in vivo rat abdominal aorta model. Five groups of vascular grafts (each *n* = 6), which included (1) rat allogenic aorta (RAA); (2) decellularized RAA (DRAA); (3) DRAA with S1P (DRAA/S1P); (4) DRAA with EC recellularization (DRAA/EC); and (5) DRAA with S1P and EC recellularization (DRAA/EC/S1P), were implanted into the rat infra-renal aorta for 14 days. The graft patency, endothelization and tissue structure were evaluated and compared.

## 2. Results

### 2.1. Characteristics of DRAA

**Figure 1:**
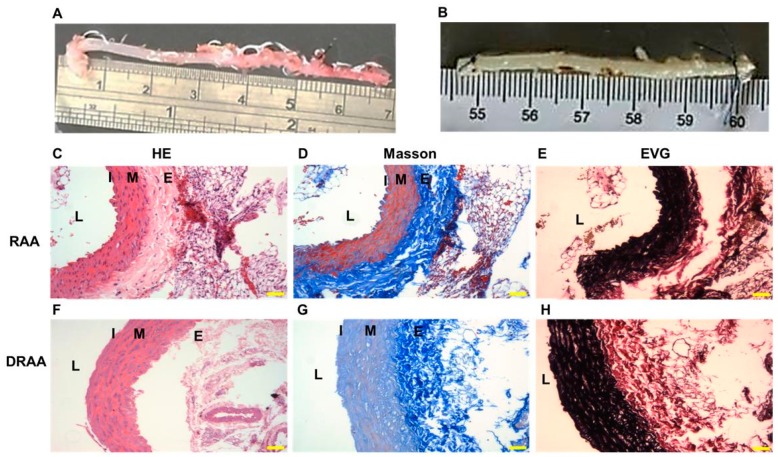
Gross appearance and histology of RAA and DRAA. (**A**,**B**) Gross appearance of the grafts ((**A**) RAA, (**B**): DRAA). (**C**–**E**) Histology of RAA (**C**) H&E stain, 200×; scale bar = 5 µm; (**D**), Masson’s trichrome stain; (**E**) Elastin Van Gieson stain). (**F**–**H**) Histology of DRAA (**E**,**F**,**H**) stain, 200×, scale bar = 5 µm; (**G**) Masson’s trichrome stain; (**H**) Elastin Van Gieson stain). (**L**) Lumen; (**I**) Intima; (**M**) Media; (**E**) Adventitia.

Compared with the gross view of RAA (Figure 1A), DRAA turned slightly transparent (Figure 1B), which shows that some components were removed after decellularization. Hematoxylin and eosin (H&E) staining showed that most of the cellular components at the luminal side were removed, although nuclear stains were still noted at the media layer, while the layer structure was preserved (Figure 1C,F). Masson’s trichrome staining demonstrated that the collagen in the decellularized vessel was maintained at the media and adventitia (Figure 1D,G). Elastin Van Gieson (EVG) staining also showed preservation of the elastin fibers in the media layer after decellularization (Figure 1E,H).

### 2.2. Characteristics of Rat ECs

**Figure 2:**
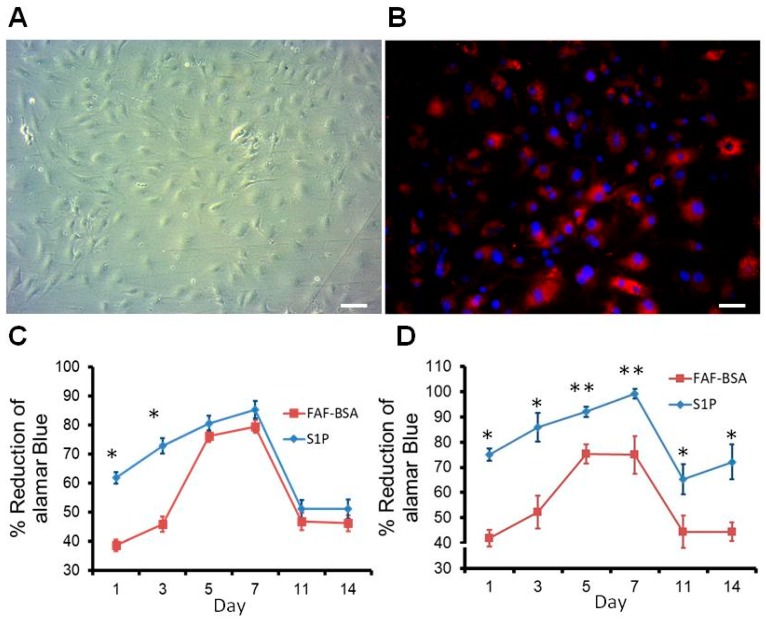
The morphology and characteristics of rat ECs. (**A**) Rat ECs in culture were viewed under bright field; (**B**) Cells that took up Dil-Ac-LDL from the medium appeared as red fluorescence, which indicated that the primary culture of cells were ECs. (DAPI as blue fluorescence), Magnification, 200×; Scale bar = 50 µm. (**C**,**D**)The proliferation of rat endothelial cells with S1P on Petri dishes (**C**) and decellularized grafts (**D**). * *p* < 0.05, ** *p* < 0.01.

Endothelial cells derived from the rat abdominal aorta grew in confluent monolayers after 12 days in culture. These cells were homogenous, closely opposed, and polygonal with indistinct cell borders by phase contrast microscopy (Figure 2A). To distinguish the fibroblasts and smooth muscle cells that might be mixed in the cell population, Dil-Ac-LDL, which binds to a receptor on the surface of vascular endothelial cells, was utilized to identify the purity of ECs from primary cultures (Figure 2B). Ten fields under a microscope at 100× magnification were taken, and the number of red fluorescent cells was counted. The Dil-Ac-LDL uptake results demonstrated that the purity of the ECs was more than 95% (Figure 2B). Although some studies revealed that differentiated ECs rapidly lose their specialized characteristics when isolated from their natural tissue microenvironment, it was detected on gene level. ECs (passage 8) in our study were proved to be CD31 positive. (Supplementary Materials Figure S1) It could uptake Dil-Ac-LDL and forming tube in vitro (data not shown) before doing the following experiments.

### 2.3. Proliferation Effect of S1P on Rat ECs on DRAA

Cell proliferation on Petri dishes and scaffolds was assessed by the results of the Alamar Blue assay. Alamar Blue is widely used for time-course proliferation assays due to its low cytotoxicity to cells [11]. The growing cells cause a chemical reduction of the dye from nonfluorescent blue to fluorescent red. Therefore, the percentage of reduction represents the number of alive cells. The viabilities of the ECs grown on a plastic surface and a decellularized matrix were evaluated using the Alamar Blue assay according to the standard protocol described in Section 3.3. The cells were incubated in the Petri dish or DHUV patch during the period of experiment without subculture. The data displayed the growth curve of ECs on Petri dishes for 14 days. After one to three days of incubation, the cells treated with S1P grew faster than the FAF-BSA control. The number of cells reached the highest point from the fifth to seventh day and decreased after that (Figure 2C). ECs grown on the scaffold exhibited a similar pattern as those grown on dish. Nevertheless, the number of S1P-treated ECs grown on DRAA was significantly higher than the number grown from ECs supplied with FAF-BSA from Day 1 to Day 14 (*p* < 0.05) (Figure 2D). Although a significant decrease in cell number on days 11 and 14 (peeling off of the confluent cells), the results indicates that S1P caused the increased viability of S1P-treated ECs compared to the FAF-BSA cells. Therefore, we have proved that rat ECs could maintain cell viability on the Petri dishes and DRAA from Day 1 to Day 11. S1P could further increase cell viability both on the Petri dish and DRAA from Day 1 to Day 14.

### 2.4. S1P Promotes SDC-1 Expression on Rat ECs

**Figure 3:**
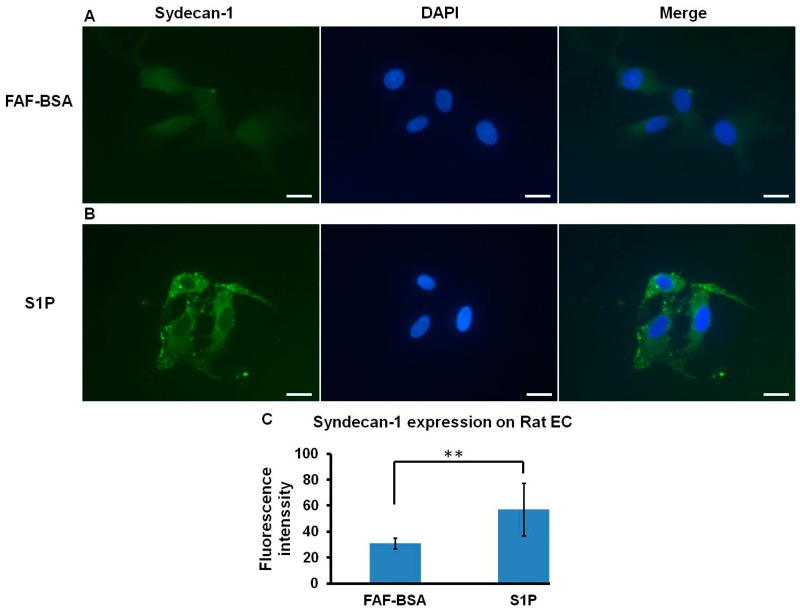
Syndecan-1 expression on rat ECs treated with S1P. In ECs treated with 1 µM S1P (**Panel B**), the expression of Syndecan-1 increased relative to that of FAF-BSA vehicle control (**Panel A**) (Left column: syndecan-1, middle column: DAPI, right column: merge; Magnification, 400×; scale bar = 50 µm); (**Panel C**), Fluorescence intensity of syndecan-1 expression on rat ECs. ** *p* < 0.01.

The expression of SDC-1 could readily be observed on rat ECs (Figure 3A) under FAF-BSA control. When ECs were treated with 1 µM S1P in the culture medium, the expression of SDC-1 on rat ECs was considerably higher than in FAF-BSA controls (Figure 3B). The fluorescence intensity of S1P-treated ECs (57.06 ± 20.2) was significantly higher than that of FAF-BSA control (30.89 ± 4.0) (*p* < 0.01) (Figure 3C). These results verified that S1P had a positive effect on SDC-1 expression on rat ECs.

### 2.5. TEVG Implantation

#### 2.5.1. Patency Rate and Animal Survival

**Figure 4:**
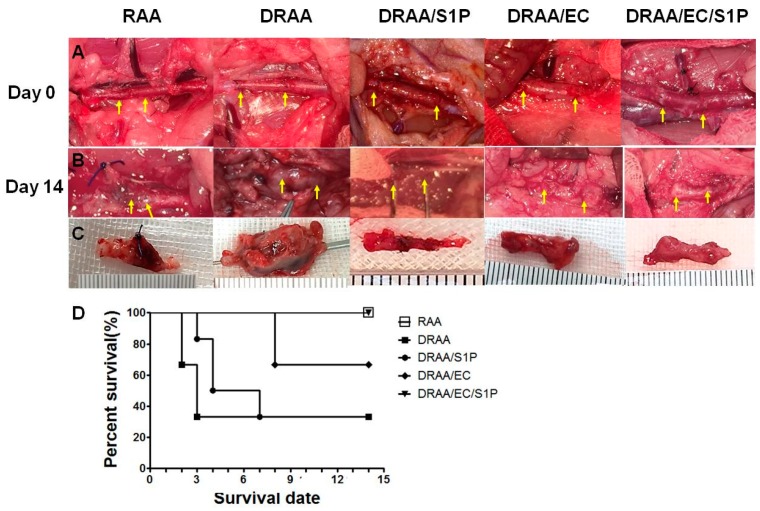
Graft implantation and survival data. (**Panel** **A**) Gross appearance of the grafts at Day 14 post-implantation (upper panel: rat abdominal aorta, arrows indicate suture lines; lower panel: explantation; left column: RAA, left-middle column: DRAA, middle column: DRAA/S1P, right-middle column: DRAA/EC, and right column: DRAA/EC/S1P). (**Panel** **B**) Gross appearance of the implanted graft at Day 14 post implantation; (**Panel** **C**) Gross appearance of the implanted graft at Day 14; (**Panel** **D**) The survival curve of the grafts.

All implanted grafts in survived rats at 14 days were evaluated; necropsy was done for all dead rats. In this model, acute thrombosis was the major cause of death within 0–3 days postoperatively. Late death (>7–14 days) could be due to stenosis which finally lead to occlusion or longer, aneurysmal rupture (>14 days). In our pretest of over 20 rats that received autograft implantation, there was no thrombosis or occlusion. Thus, we believed the surgical procedure was well established. No anastomosis related vessel occlusion (such as back-wall suture) was noted at anastomotic sites of the explanted grafts. After surgery, only one of six rats were alive after 14 days in the DRAA group, compared with two and four of six in the DRAA/S1P and DRAA/EC groups, respectively. In contrast, all six rats survived 14 days after surgery in the RAA and DRAA/EC/S1P groups (Table 1). Figure 4A and 4B show implanted vessels on Day 0 and Day 14, and Figure 4C presents the explanted vascular tissues of the five groups. At Day 14, DRAA showed aneurysmal dilatation (DRAA, Panels B and C; diameter increase in 3–4×) but no any rats died of ruptured aneurysm. The patency rate of RAA and DRAA/EC/S1P both achieved 100% during the 14-day follow-up. The patency rates of DRAA, DRAA/S1P and DRAA/EC were only 50%, 40% and 63%, respectively. The dead rats did not have aneurysm. The five rats in DRAA group died due to acute thrombosis within 0–3 days postoperatively. At this time-point, the explanted grafts did not show dilatation. The similar findings were noted in other groups. Only thrombosis and lumen occlusion was noted in the explanted grafts of dead rats. The statistical survival curve is shown in Figure 4D. Rats that received implantation of RAA and DRAA/EC/S1P had a 100% survival rate within 14 days.

#### 2.5.2. Histomorphology of Explanted Vessels

**Figure 5:**
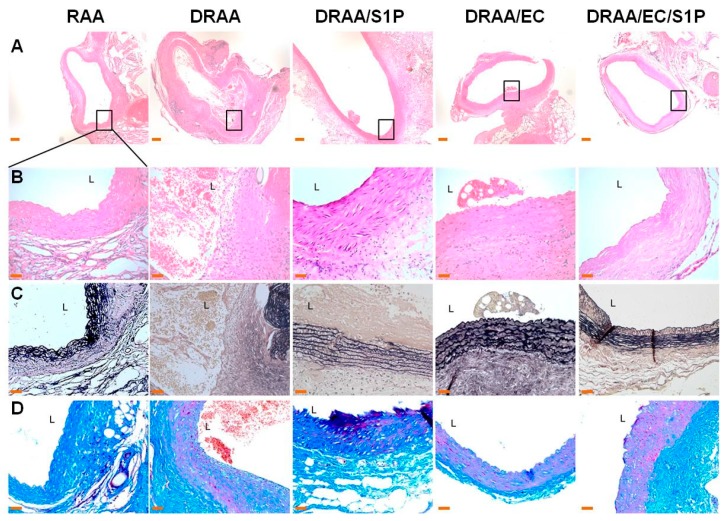
Histological stains of explanted grafts at Day 14. (**Panels** **A**–**D**) show rat abdominal aorta stained using H&E ((**A**): Magnification, 40×, (**B**): Magnification, 400×), EVG ((**C**): Magnification, 200×), and Masson’s trichrome (MT) ((**D**): Magnification, 200×). Stained sections show distributions of elastin (black in EVG), smooth muscle (red in MT), and collagen (blue in MT) (left column: RAA, left-middle column: DRAA, middle column: DRAA/S1P, right-middle column: DRAA/EC, and right column: DRAA/EC/S1P). The scale bar of A is 20 µm, and those of (**B**,**C**) and D are 5 µm. L, Lumen. *n* = 6 for RAA & DRAA/EC/S1P, *n* = 4 for DRAA/EC, *n* = 2 for DRAA/S1P and *n* = 1 for DRAA.

Because the grafts of dead rats were only checked grossly and the thrombosis was viewed inside the vessel lumen (data not shown), these samples were not processed to histology or IHC staining because of the short survival days. Hence, the number of animal for H&E, EVG and Masson’ trichrome and IHC staining in each group was *n* =6 for RAA & DRAA/EC/S1P, *n* = 4 for DRAA/EC, *n* = 2 for DRAA/S1P and *n* = 1 for DRAA. At Day 14 post-implantation, RAA was essentially normal with intact intima, media and adventitia. No thrombosis formation was noted in the lumen. The elastin fibers remained in a layered structure in the media. No obvious surrounding fibrotic tissue was noted. In DRAA, there was endothelial damage and thrombotic plaque formation on the luminal side. The destruction of media with elastin-layered effects was noted. Fibrosis surroundings were also noted. In DRAA/S1P, the abdominal aorta was generally intact, while focal intimal thrombotic plaque was found with fibrin clots and no endothelial lining. The layered elastin structure and collagen fibers remained intact in the media. There was obvious cellular infiltration at the adventitia. In DRAA/EC, although there was no obvious endothelial lining, fewer thrombotic plaques or fibrin clots were noted in the lumen. In DRAA/EC/S1P, there was no obvious thrombotic plaque formation in the lumen, and endothelial cells were found. The elastin and collagen fibers remained well-organized in the media. More cellular infiltration at the adventitia was noted. There was no obvious surrounding fibrotic tissue (Figure 5).

#### 2.5.3. Endothelialization of Implanted Vessels

**Figure 6:**
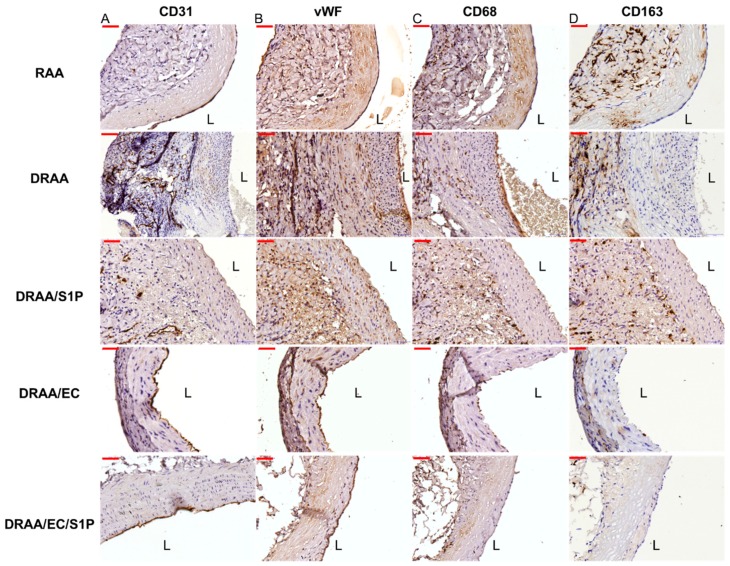
IHC staining of the explanted grafts at Day 14. Rat abdominal aorta stained using (**Panel A**) CD31; (**Panel B**), vWF; (**Panel C**), CD68; and (**Panel D**), CD163. (left column: RAA, left-middle column: DRAA, middle column: DRAA/S1P, right-middle column: DRAA/EC, and right column: DRAA/EC/S1P).Magnification all 400×, scale bar 50 µm. L, Lumen. *n* = 6 for RAA & DRAA/EC/S1P, *n* = 4 for DRAA/EC, *n* = 2 for DRAA/S1P and *n* =1 for DRAA.

At Day 14 post-implantation, the CD31 and vWF stains highlighted the luminal CD31- or vWF-positive endothelial cells in the implanted vascular grafts of the RAA, DRAA/EC and DRAA/EC/S1P groups, while the vascular grafts of the DRAA and DRAA/S1P groups were devoid of endothelial cells. The results were confirmed with H&E stains (Figure 6A,B).

#### 2.5.4. Macrophage Infiltration of Implanted Vessels

At Day 14 post-implantation, dense infiltrates of CD68- or CD163-positive histiocytes were present mainly in the tunica adventitia of the RAA, DRAA and DRAA/S1P groups, while the cellularity of CD68- or CD163-positive histiocytes in the DRAA/EC and DRAA/EC/S1P groups was scant (Figure 6C,D).

## 3. Materials and Methods

**Figure 7:**
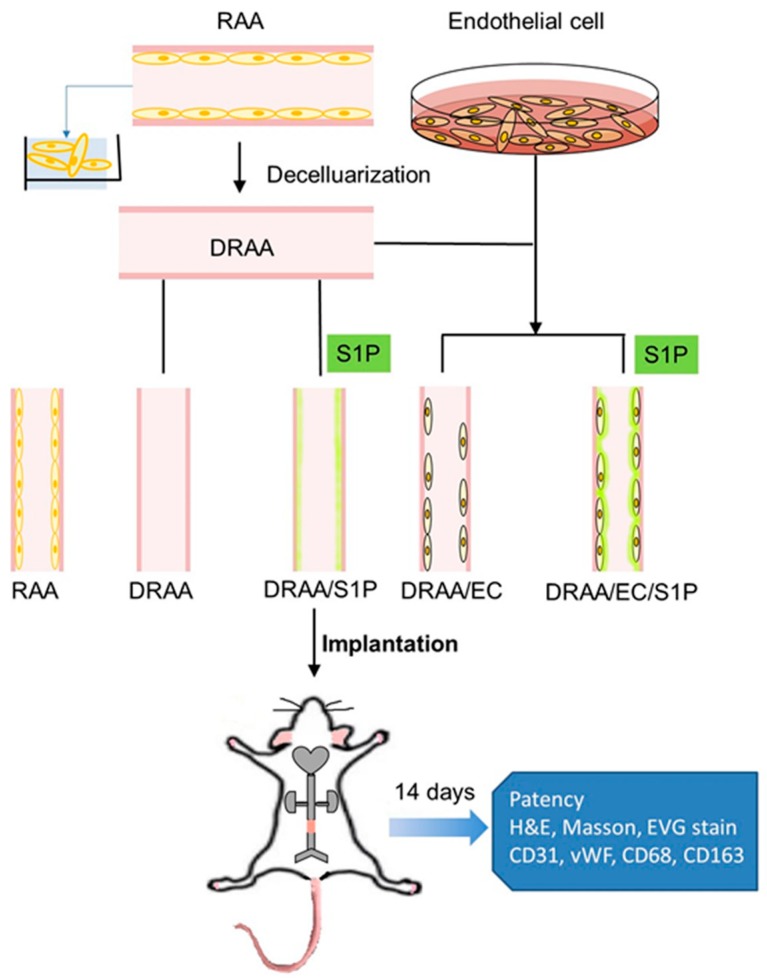
Schematic diagram of the experiment. The TEBV was constructed by decellularized rat abdominal aorta and rat ECs. S1P was added for 48 h to the ECs while seeding on the scaffold. To evaluate the anti-thrombotic effect of S1P on this small caliber vascular, the RAA, DRAA, DRAA with S1P and DRAA seeded by EC only were implanted into rat aorta separately (*n* = 6).

The schematic diagram of the experiment is shown in Figure 7. The animal study was approved by the institutional review board and institutional animal care/use committee (Approval number 2016–228, approval date 14 December 2016. Taipei Veterans General Hospital, Taiwan). The study was carried out according to National Institutes of Health guide for the care and use of Laboratory animals. Animal care was performed according to the principle of Arrive guideline. All animals were kept in oxygen-supplied breeding cages. All rats had free access to deionized water and food.

### 3.1. Preparation and Storage of Native and Decellularized Rat Aortas

The rats were anesthetized with an intraperitoneal injection of 50 mg/kg body weight of Zoletil 50 (Virbac, France) before harvesting the aorta. The aortas below the diaphragm were approached through an abdominal midline incision, and the visceral was lateralized. The aorta was identified and meticulously separated from the inferior vena cava in the retroperitoneal space. Then, an intracardiac infusion of Lactated Ringer’s solution (Y F CHEMICAL CORP., New Taipei City, Taiwan) with 10 U/mL heparin (China Biotech Corporation, Taichung, Taiwan) was performed to flush blood out of the vessel. After clamping the proximal end and ligating the branches, the aorta was harvested distally to the bifurcation. The aortas were then immersed in antibiotic cocktails composed of 250 µg/mL cefuroxime, 200 µg/mL ciprofloxacin, 80 µg/mL gentamicin, 50 µg/mL vancomycin, 1000 units/mL colistin, and 200 µg/mL amphotericin B for 24 h and were preserved at 4 °C, later either used for decellularization or EC culture. Decellularization was started with a 2-day incubation using 0.1% sodium dodecyl sulfate (SDS; Sigma-Aldrich, St. Louis, MO, USA), followed by 2 days of washing with phosphate-buffered saline (PBS; pH 7.4; Gibco, Carlsbad, CA, USA). Medium 199 (Gibco, Grand Island, NY, USA) containing 20% fetal bovine serum (FBS; Gibco, Grand Island, NY, USA) was used for two days to remove remaining DNA, and the vessels were subsequently washed with PBS. The decellularization steps were performed at 37 °C on a shaker with high-speed agitation under sterile conditions. Approximately one-cm segments of native and decellularized aortas were also fixed in 4% formaldehyde for 24 h and embedded in paraffin. Then, 4 μm-thick transverse sections of the aortas underwent H&E, Masson’s trichrome and EVG staining. The remaining native and decellularized aortas were disinfected in antibiotic cocktails at 4 °C or cryopreserved in liquid nitrogen for further applications.

### 3.2. Primary Culture, Maintenance and Characterization of Rat EC

The rat aortas were washed immediately with PBS containing 150 µg/mL EDTA after recovery from the body to remove the blood. The fresh rat aortas were cut off longitudinally using sterile scissors, then the luminal surface was attached to a 10-cm culture dish with PBS containing 0.5 µg/mL type I collagenase (Sigma-Aldrich, St. Louis, MO, USA). After incubating at 37 °C for 30 min, detached ECs from the intimal layer were collected and centrifuged for 5 min at 2000 rpm. The supernatant was discarded, and the cell pellet was resuspended in endothelial cell growth medium (EGM; Cell application, CA, USA) supplemented with 10% FBS and 100 U/mL penicillin streptomycin. Then, the cells were cultured in 10-cm dishes and the medium was changed to fresh medium every two days. The cells were passaged weekly and subcultured after trypsinization. Usually, it took 2–3 weeks for ECs to proliferate in a normal pace. Thus, the expanded cultures were maintained in culture for eight to 12 weeks totally, and passages five to eight were used in the experiments. The ECs applied in the experiments were verificated by detached in non-enzymatic cell dissociation medium (Sigma-Aldrich, St. Louis, MO, USA) to preserve the surface protein and incubated for 30 min at 4 °C with anti-CD31-FITC (Abcam, Cambridge, UK) according to manufacturer’s instruction. Fluorescence analysis was performed using a flow cytometer, FACSDiva and FlowJo software (Becton Dickinson). ECs were further were incubated with l,l′-dioctadecyl-l-3,3,3′,3′-tetramethylindocarbocyanine perchlorate (DiI-ac-LDL, 10mg/mL; Molecular Probes) at 37 °C for 4h. A ZEISS inverted microscope fluorescent microscope was used to take picture. The tube formation assay was also processed to prove the function of ECs.

### 3.3. Proliferation Effect of S1P on Rat ECs

The viability and proliferation of ECs with or without S1P (Avanti Polar Lipids, AL, USA) and solvent control, which as 0.1% fatty-acid free bovine serum albumin (FAF-BSA; Sigma-Aldrich, St. Louis, MO, USA), on the dishes and DRAA (each *n* = 3) were measured by the alamarBlue assay (Invitrogen, MD, USA) according to the manufacturer’s instructions. In brief, the cells that were in the log-phase growth stage were harvested and seeded in a six-well plate directly or on the decellularized rat aortas at a density of 5 × 105 per well or 1 cm × 1 cm scaffold (Day 0). The cells were cultured in culture medium at 37 °C in a 5% CO2 environment for 24 h (Day 1). Then, the medium was replaced with new medium containing 10% alamarBlue dye, and the cells were incubated for 4 h. For absorbance measurements, 100 µL of medium was transferred into a flat-bottom 96-well plate, and the absorbance at wavelengths of 570 nm and 600 nm were measured. The assay was also performed at Day 3, 5, 7, 9, 11 and 14. The cell proliferation was presented as the % Reduction of alamarBlue following the equation below and use Molar Extinction Coefficient.

% Reduction of alamarBlue = (O2 × A1) − (O1 × A2) × 100/(R1 × N2) − (R2 × N1)

O1 = Molar Extinction Coefficient of OXIDIZED alamarBlue at 570 nm is 80586
O2 = Molar Extinction Coefficient of OXIDIZED alamarBlue at 600 nm is 117216
R1 = Molar Extinction Coefficient of REDUCED alamarBlue at 570 nm is 155677
R2 = Molar Extinction Coefficient of REDUCED alamarBlue at 600 nm is 80586
A1 = Absorbance value of test wells at 570 nm
A2 = Absorbance value of test wells at 600 nm
N1 = Absorbance value of Negative Control well at 570 nm
N2 = Absorbance value of Negative Control well at 600 nm

### 3.4. Effect of S1P on SDC1 Expression in Rat ECs

Rat ECs were prepared as described above and seeded on ethanol-disinfected 18-mm round coverslips (Bioman Scientific Co., Ltd., New Taipei City, Taiwan) and placed in 12-well plates. A total of 2 × 10^6^ cells/per well were applied and incubated at 37 °C, 5% CO_2_ for 1 h. Then, FBS-free EGM was added and the culture was incubated overnight. Afterwards, medium containing 1 µM S1P and 0.1% FAF-BSA was added and incubated for 6 h. The coverslips were stained with anti-SDC-1 (Abcam, Cambridge, UK) using Alexa Fluor 647 goat anti-rabbit secondary antibody (Invitrogen, Carlsbad, CA, USA). For immunofluorescence staining, the cells were fixed with 2% paraformaldehyde and blocked with 5% BSA (BIOMAN, Taiwan) in PBS solution. Glycocalyx shedding was imaged with a Zeiss AxioPlan 2 fluorescence microscope (400×) and quantified with ImageJ. 10 fields from each treatment were randomly chosen to count the fluorescence of the cells. The data was statistical analyzed by one-way ANOVA.

### 3.5. Construction of TEVGs

TEVGs were constructed by decellularized rat allogenic aortas (DRAA), rat ECs and S1P by the static seeding method. The cultured ECs at passages five to eight were harvested and diluted with 1 mL of EGM culture medium and 1 μM S1P. The cell suspension (1.0 × 10^6^ cells/mL, 1 mL) was injected into the lumen of decellularized rat aortas by a 1-mL syringe to facilitate dilatation of the lumen (length, 1 cm; inner diameter, ~1–1.5 mm), and the aorta was then placed in a 10-cm dish with the two ends clamped by microvessel clamps. The aorta was immersed in the culture medium for 6 h at 37 °C while rotating 360° around its longitudinal axis. Another cell suspension was then added in the same manner, and the graft was rotated 90° around its longitudinal axis. After repeating this procedure four times (total seeded cells, 4.0 × 10^6^ cells), the graft was incubated for another 24 h to ensure complete cell attachment. After four days of static incubation in EGM, the cell suspension in the cultured tube was renewed with fresh medium and incubated for another 24 h to ensure complete cell attachment and for animal study later. The DRAA/EC grafts were generated without adding 1 μM S1P into the culture medium of ECs, and the cells were subsequently seeded on DRAA with the same approach described above. The DRAA/S1P grafts were submerged in EC culture medium with 1 μM S1P for two days, followed by washing with Medium 199 (Gibco, USA) three times. The grafts were kept in fresh Medium 199 until use.

### 3.6. Rat Abdominal Aorta Interposition Graft Model

Thirty female SD rats (12 weeks old, BioLASCO, Yilan, Taiwan) were divided into five groups in terms of the implanted vessels (*n* = 6): Group 1: RAA; Group 2: DRAA; Group 3: DRAA/S1P; Group 4: DRAA/EC; and Group 5: DRAA/ EC/S1P. The SD rats were anesthetized with an intraperitoneal injection of 50 mg/kg body weight of Zoletil 50 (Virbac, Carros cedex, France). Briefly, the anesthetized rat was placed in a supine position over a warm pad. After shaving and sterilization, a midline laparotomy was performed. Then, the abdominal aorta between the infra-renal artery and iliac artery bifurcation was explored after lateralizing the intestine and opening the retroperitoneal fascia. The aorta was carefully dissected from the inferior vena cava, and the side branches were ligated. After clamping proximally and distally, and 1-cm aorta segment was replaced with the above five group vessels. The end-to-end anastomosis was performed with 9–0 nylon interrupted sutures. No anticoagulation or anti-platelet drugs were given perioperatively. After the clamps were released, the hemostasis was checked, the visceral vessels were put back, and the wound was closed in layers. The rats recovered from anesthesia in a separate cage, where they received food and water ad libitum. In our pretest of over 20 rats which received autograft implantation, there were no thrombosis occlusion. Thus, we believed the surgical procedure was well-established. Female rats were purchased because in our experience, female rats are relative easily handled. There was neither particular meaning for only using female rats nor expecting gender-specific differences in the results in this study.

All implanted vessels and TEVGs were expected for histological examination 14 days after implantation if survived. Otherwise, necropsy was done for dead rats. The procedure of explantation was the same as above under anesthesia. All the explanted grafts were fixed with 4% formaldehyde solution for 24 h and dehydrated with a series of ethanol solutions. The samples were then embedded in paraffin. Four µm-thick transverse sections from the midportion segments of the grafts were stained with HE, EVG and Masson’s trichrome staining.

To evaluate graft re-endothelialization and inflammation after implantation, immunohistochemistry was performed on an automated IHC staining system named the Bond-MAX system (Leica Biosystems, Melbourne, Australia) with the Bond Polymer Refine Detection Kit (Leica, UK). First, formaldehyde-fixed paraffin-embedded tissue sections were pretreated using heat-mediated antigen retrieval with sodium citrate buffer (pH 6, epitope retrieval solution 1) for 30 min. Then, the slides were incubated with anti-CD31 (Abcam, UK, dilution 1:100), anti-vWF (Abcam, UK, dilution 1:1800), anti-CD63 (Abcam, UK, dilution 1:100) and anti-CD168 (Abcam, UK, dilution 1:100) for 60 min at room temperature, followed by secondary HRP antibodies that were part of an HRP-conjugated compact polymer system (rabbit anti-mouse IgG and anti-rabbit IgG-Poly-HRP). After blocking with peroxide for 5 min, DAB was used as the chromogen on the stained sections. Finally, hematoxylin for nuclear counterstaining was processed, and sections were mounted with DPX (Sigma-Aldrich, MO USA). Images were captured with a ZEISS inverted microscope.

### 3.7. Statistical Analysis

All cell experiments were repeated at least three times, and the data are shown as the mean ± SD. The Mann–Whitney U test was applied for statistical analysis between experimental groups where *p* < 0.05 was considered significant. The Kruskal–Wallis test with post hoc Mann–Whitney U test was used to compare data for more than two groups where *p* < 0.05 was considered significant. Statistical analysis was performed using IBM SPSS Statistics 19 (version 19; SPSS, Chicago, Illinois).

## 4. Discussion

Early confluent endothelialization at the luminal surface of the decellularized vascular grafts is the critical step for graft patency in vivo [12]. Intact endothelialization could avoid exposure of the extracellular matrix component, smooth the luminal surface and play a role in the thrombosis-resistant interface with the blood, thereby avoiding platelet aggregation, the coagulation cascade and clot formation. Exposure of the matrix underlying the endothelial monolayer may also bring in remodeling of the matrix and stimulating inflammatory cells, which results in severe intimal hyperplasia that may lead to stenosis of the grafts [13].

Using growth factors or biomolecules to overcome thrombus formation and EC recruitment is a strategy for improving the endothelialization of decellularized vascular grafts in vivo. The growth factors include heparin, vascular endothelium growth factor (VEGF), granulocyte-colony stimulating factor (G-CSF), brain-derived growth factor (BDGF), cysteine-rich protein (CYR61; CCN1) and stromal cell-derived factor (SDF-1α), were beneficial for graft patency, an effect of early endothelialization, and perhaps also for graft remodeling [14,15,16,17,18,19,20]. Recellularization before implantation is another route to improve endothelialization of decellularized vascular grafts in vivo. EC, SMCs or EPCs may be feasible for use in recellularization before implantation [21,22]. Other cell resources, such as mesenchymal stem cells (MSC), pericytes, circulatory or adipose tissue progenitor cells and induced pluripotent stem (iPS) cells, also show great potential for use in tissue engineering applications [23,24].

For application of S1P in reconstitution of TEVGs, it has been shown that S1P-releasing materials produced a strong angiogenic response in the chick chorioallantoic membrane assay and increased the migration speeds of human umbilical vein endothelial cells attached to S1P-releasing hydrogels containing linear RGD peptides [25]. S1P also significantly increased platelet-related angiogenesis and induced tube formation in HUVECs grown on Matrigel [26]. In addition, S1P induced VEGF-C expression and PECAM-1 phosphorylation in endothelial cells through the MMP2/FGF-1/FGFR-1 pathway and c-Src activation, respectively [26,27]. There was also a synergistic effect between S1P, VEGF and fluid flow [28].

For the recellularization of decellularized vascular grafts, the stability of adhesive ECs under fluid shear stress is crucial once implanted. S1P promotes endothelial cell migration in vitro and may potentially impact the endothelialization of implanted biomaterials [28]. The strength of the interaction of endothelial cells with a substrate is also critical for the motility of the cells [13]. Optimal attachment forces also allow the cells to pull themselves forward and release their rear edges. The strength of cell adhesion and migratory ability of ECs on different biomaterials have been found to be related to S1P concentration [29,30]. Moreover, S1P also plays a role in maintaining EC barrier function through the glycocalyx layer, which mainly comprises heparan sulfate, chondroitin sulfate, and hyaluronic acid, at the blood–tissue interface. This layer of glycocalyx that covers ECs exerts a series of biological functions, such as maintaining vascular permeability and signal transmission [31]. A study using rat mesenterial microvessels showed that S1P played a crucial role in stabilizing the glycocalyx of ECs and maintaining normal vascular permeability [32].

Stabilization of the glycocalyx of ECs is important to prevent thrombus formation while using ECs for the recellularization of decellularized vascular grafts. In the glycocalyx, glypican-1 and SDC-1 are two major heparan sulfate proteoglycans on ECs with potent anti-thrombotic action; S1P contributes to the protection of the glycocalyx via the S1P1 G-protein coupled receptor (GPCR) [33]. Shedded SDC-1 could aggregate with vWF, fibrin, fibronectin, and bacteria and lead to thrombi that occlude the lumen of vessels by activating platelets and leukocytes [34]. Our previous in vitro study also demonstrated that S1P enhanced the attachment of ECs and EPCs to decellularized human umbilical veins. Furthermore, S1P promotes SDC-1 expression in ECs on decellularized human umbilical veins, and SDC-1 expression correlated with a significant reduction in platelet aggregation [10].

The current study further demonstrated the advantages of EC-plus-S1P-recellularized vascular grafts over EC-only or S1P-only grafts with regard to the 14-day patency in a rat aorta model. EC-plus-S1P grafts also had superior results than allogenic grafts, at least in the acute stage. Patent grafts revealed confluent endothelial lining, which was demonstrated by CD31/vWF staining, and only a mild inflammatory reaction comparable to that of allografts. Patency may be related to endothelialization prior to implantation or the anti-thrombotic effect of S1P on cell-seeded decellularized scaffolds after in vivo perfusion. The increased patency and endothelialization in EC-recellularized grafts over S1P-added grafts also demonstrated the importance of recellularization. Incomplete endothelialization is related to higher thrombogenicity and may imply higher obstruction rates. Incomplete EC coverage could be related to an initial low cell concentration for seeding, a low cell attachment rate and attached cell detach during the implantation process [35,36,37]. Although we did not know if the cell density we applied in recellularization was sufficient for complete coverage, S1P did play a role in securing EC attachment, anchoring cells and maintaining cell stability on the scaffolds before and during implantation. Furthermore, histological examinations of EC-plus-S1P-recellularized vascular grafts revealed an extracellular matrix consisting of well-preserved, normally arranged collagen and elastin fibers. The presence of a layered structure with wavy fibers also correlated with the mechanical properties that withstand pulsatile blood pressure. Existing endothelial cells may also regulate matrix synthesis by secreting extracellular matrix products, cytokines, and chemokines [38] The improved results in EC-plus-S1P seeded vascular grafts over only S1P-added grafts also showed that the anti-thrombotic effect of S1P was through coupling with ECs in vivo.

In brief, this proof-of-concept study showed the feasibility of lipid biomolecules, S1P with EC recellularized small-caliber tissue engineered vascular grafts in vivo. The scenario captured the principle of tissue engineering which incorporated cell, signal and scaffold to reconstitute tubular tissue in vitro and then was transplanted vivo. S1P increased proliferation of ECs on decellularized vascular grafts and enhanced anti-thrombogenicity by augmentation of SDC-1 expression. The reconstituted TEVGs expressed patency and endothelialization under arterial flow in rat model within 14-day follow-up.

There were limitations in the study. First, the relatively low animal number in each group could bias the interpretation of results. Second, the follow-up period was only 14 days, and only an acute reaction was revealed. Long-term evaluation for chronic inflammation and tissue remodeling is needed. Third, we used only female rats in animal study. Whether the animal gender would affect the results needs further study. Fourth, the current study only focused on the morphometric measurements of graft structure and as S1P has been shown to critically affect vascular function, investigation of the functional properties of the induced endothelial lining in TEVGs is warranted. Fifth this is a small animal model, and whether the results can be translated to humans is unknown. Future large animal studies are necessary.

## 5. Conclusions

Our results demonstrated that EC-plus-S1P-recellularized decellularized allogenic vascular grafts have an acceptable patency rate and endothelialization in a rat aorta implantation model. The study suggests that S1P is an effective additive capable of decreasing the thrombotic risk in decellularized vascular grafts seeded with endothelial-lineage cells. Thus, our report may provide a novel direction for the generation of tissue-engineered blood vessels with enhanced antithrombogenic properties that are especially important for small-caliber vascular grafts.

## Figures and Tables

**Table 1:** The survival date of animals after surgery (days).

No	RAA	DRAA	DRAA/S1P	DRAA/EC	DRAA/EC/S1P
1	14	2	7	14	14
2	14	14	3	14	14
3	14	3	14	14	14
4	14	0	4	8	14
5	14	0	14	8	14
6	14	0	4	14	14

## Supplementary Materials

Supplementary materials can be found at https://www.mdpi.com/1422-0067/20/7/1641/s1.
