# Supplementary material for: Sphingosine-1-phosphate in Endothelial Cell Recellularization Improves Patency and Endothelialization of Decellularized Vascular Grafts In Vivo
Source: Int J Mol Sci. 2019 Apr 2;20(7):1641. doi: 10.3390/ijms20071641 (PMC6480112; doi:10.3390/ijms20071641)
Supplement: Supplementary file 1 [file ijms-20-01641-s001.pdf]

International Journal of
Molecular Sciences

MDPI

Comprehensive list of changes

This is the *last chance* to make textual changes to the manuscript.

Line	Before proof reading	After Proof Reading
22-23	Taipei Zip code, Taiwan;	Taipei 11217, Taiwan;
25	Correspondence jenherlu@gmail.com; Tel: 886-2-8757542; Fax: 886-2-28757019	Correspondence jenherlu@gmail.com; Tel: 886-2-77370020; Fax: 886-2-77370023
60	compliance matching, biodegradability and tissue remodeling [4-5].	compliance matching, biodegradability and tissue remodeling. [4-5]
96-67	H&E staining showed that most of the cellular components at the luminal side were removed	Hematoxylin and eosin (H&E) staining showed that most of the cellular components at the luminal side were removed
100	EVG staining also showed preservation of the elastin fibers in the media layer after decellularization (Figure 1E and 1H).	Elastin Van Gieson (EVG) staining also showed preservation of the elastin fibers in the media layer after decellularization (Figure 1E and 1H).
107	L, Lumen. I, Intima. M, Media. E, Adventitia.	L, Lumen; I, Intima; M, Media; E, Adventitia.
119	ECs (passage 8) in our study was proved to be CD31 positive.	ECs (passage 8) in our study were proved to be CD31 positive.
128	2.3.Proliferation Rffect of S1P on Rat ECs on DRAA	2.3. Proliferation Effect of S1P on Rat ECs on DRAA.
151		Add * $p < 0.05$, ** $p < 0.01$.
156	the expression of SDC-1 on rat ECs was considerably higher than in FAF-BSA controls. (Figure 3B).	the expression of SDC-1 on rat ECs was considerably higher than in FAF-BSA controls (Figure 3B).
157	The fluorescence intensity of S1P-treated ECs (57.06 ± 20.2) was significantly higher than that of FAF-BSA control (30.89 ± 4.0)	The fluorescence intensity of S1P-treated ECs (57.06 ± 20.2) was significantly higher than that of FAF-BSA control (30.89 ± 4.0)
158	These results verified that S1P had a positive effect on SDC-1 expression in rat ECs.	These results verified that S1P had a positive effect on SDC-1 expression on rat ECs.
161	Syndecan-1 expression in rat ECs treated with S1P.	Syndecan-1 expression on rat ECs treated with S1P.
162	the expression of Syndecan-1 increased relative to that of FAF-BSA vehicle controls	the expression of Syndecan-1 increased relative to that of FAF-BSA vehicle control
164	**,p< 0.01.	**p< 0.01.
168	In this model, acute thrombosis is the major cause of death within 0–3 days postoperatively.	In this model, acute thrombosis was the major

		cause of death within 0–3 days postoperatively.
171	In our pretest of over 20 rats that received autograft implantation, there were no thrombosis or occlusion	In our pretest of over 20 rats that received autograft implantation, there was no thrombosis or occlusion.
189	The dead rats didn't all have aneurysm.	The dead rats did not have aneurysm.
191	At this time-point, the explanted grafts didn't show dilatation.	At this time-point, the explanted grafts did not show dilatation.
202	Panel C) Gross appearance of the implanted graft at Day 0;	Panel C) Gross appearance of the implanted graft at Day 14;
203	2.5.2. Histomorphology of Explanted Vessels (Figure 5)	2.5.2. Histomorphology of Explanted Vessels
205	Because the grafts of dead rats were only checked grossly and the thrombosis was viewed inside the vessel lumen (data not shown). These samples were not processed to histology or IHC staining because of the short survival days.	Because the grafts of dead rats were only checked grossly and the thrombosis was viewed inside the vessel lumen (data not shown), these samples were not processed to histology or IHC staining because of the short survival days.
226	There was no obvious surrounding fibrotic tissue.	There was no obvious surrounding fibrotic tissue (Figure 5).
227	**Panels A** to **D** show rat abdominal aorta stained using hematoxylin and eosin (H&E)	**Panels A** to **D** show rat abdominal aorta stained using H&E,
229	Elastin Van Gieson (EVG) (**C**: Magnification, 200×), and Masson's trichrome (MT)	EVG (**C**: Magnification, 200×), and Masson's trichrome (MT)
234	n = 2 for DRAA/S1P and n = 1 for DRAA	n = 2 for DRAA/S1P and n = 1 for DRAA.
235	2.5.3. Endothelialization of Implanted Vessels (Figure 6)	2.5.3. Endothelialization of Implanted Vessels
239	The results were confirmed with H&E stains.	The results were confirmed with H&E stains (Figure 6 Panel A and Panel B).
240	2.5.4. Macrophage Infiltration of Implanted Vessels (Figure 6)	2.5.4. Macrophage Infiltration of Implanted Vessels
250	the cellularity of CD68- or CD163-positive histiocytes in the DRAA/EC and DRAA/EC/S1P groups was scant.	the cellularity of CD68- or CD163-positive histiocytes in the DRAA/EC and DRAA/EC/S1P groups was scant (Figure 6 Panel C and Panel D).
256	*n* = 2 for DRAA/S1P and n =1 for DRAA	*n* = 2 for DRAA/S1P and *n* =1 for DRAA.
260	*(Approval number 2016-228, Taipei Veterans General Hospital, Taiwan).*	*(Approval number 2016-228, approval date December*

		14th,2016. Taipei Veterans General Hospital, Taiwan).
275-276	Lactated Ringer's solution (Y F CHEMICAL CORP., Taiwan) with 10 U/mL heparin (China Biotech Corporation, Taiwan)	Lactated Ringer's solution (Y F CHEMICAL CORP., New Taipei City, Taiwan) with 10 U/mL heparin (China Biotech Corporation, Taichung, Taiwan)
284-285	Medium 199 (Gibco) containing 20% fetal bovine serum (FBS; Gibco, South America)	Medium 199 (Gibco, Grand Island, NY, USA) containing 20% fetal bovine serum (FBS; Gibco, Grand Island, NY, USA)
290	4 µm-thick transverse sections of the aortas underwent hematoxylin and eosin (H&E), Masson's trichrome and Elastin Van Gieson (EVG) staining.	4 µm-thick transverse sections of the aortas underwent H&E, Masson's trichrome and EVG staining.
307	anti-CD31-FITC (Abcam, UK)	anti-CD31-FITC (Abcam, Cambridge, UK)
310	DiI-acLDL	DiI-ac-LDL
341	18-mm round coverslips (Bioman Scientific Co., Ltd., Taiwan)	18-mm round coverslips (Bioman Scientific Co., Ltd., New Taipei City, Taiwan)
345	anti-SDC-1 (Abcam, UK)	anti-SDC-1 (Abcam, Cambridge, UK)
350	The data was statistical analyzed by one-way ANOVA .	The data was statistical analyzed by one-way ANOVA.
366	Medium 199 (Gibco, USA)	Medium 199
369	Thirty female SD rats (12 weeks old, BioLASCO, Taiwan)	Thirty female SD rats (12 weeks old, BioLASCO, Yilan, Taiwan)
372	Zoletil 50 (Virbac, France).	Zoletil 50 (Virbac, Carros cedex, France).
455-456	S1P contributes to the protection of the glycocalyx via the S1P1 G-protein coupled receptor (GPCR) [33].	S1P contributes to the protection of the glycocalyx via the S1P1 G-protein coupled receptor (GPCR). [33]
461	SDC-1 expression correlated with a significant reduction in platelet aggregation [10].	SDC-1 expression correlated with a significant reduction in platelet aggregation. [10]
511	W.-M. C.Methodology, C.-L. Y.Reviewing and Supervision,	W.-M. C. Methodology, C.-L. Y. Reviewing and Supervision,
513-514	**Funding:** Please add: "This research received no external funding" or "This research was funded by [name of funder] grant number [xxx]" and "The APC was funded by [XXX]". Check carefully that the details given are accurate and use the standard spelling of funding agency names at	**Funding:** This research was funded by I-MEI FOODS CO., LTD and Taipei Veterans General Hospital grant number R16001 and R16002, MOST 107-2314-B-075-030.

	https://search.crossref.org/funding, any errors may affect your future funding.	
515	**Acknowledgments:** This work was supported by Grant R16001 & R16002 from I-MEI FOODS CO., LTD and Taipei Veterans General Hospital.	**Acknowledgments:** We thank for the support of I-MEI FOODS CO., LTD to our research.
		Change all the citation notes to [1].
